# Bio-Based Epoxy Adhesives with Lignin-Based Aromatic Monophenols Replacing Bisphenol A

**DOI:** 10.3390/polym13223879

**Published:** 2021-11-10

**Authors:** Nigel Van de Velde, Saška Javornik, Tilen Sever, Danaja Štular, Matic Šobak, Žiga Štirn, Blaž Likozar, Ivan Jerman

**Affiliations:** 1 National Institute of Chemistry, Hajdrihova 19, 1001 Ljubljana, Slovenia; nigel.van.de.velde@ki.si (N.V.d.V.); saska.javornik@ki.si (S.J.); danaja.stular@ki.si (D.Š.); matic.sobak@ki.si (M.Š.); ziga.stirn@ki.si (Ž.Š.); blaz.likozar@ki.si (B.L.); 2 Steklarna Hrastnik, d. o. o., Cesta 1. maja 14, 1430 Hrastnik, Slovenia; tilen.sever@hrastnik1860.com; 3 Pulp and Paper Institute, Bogišićeva 8, 1000 Ljubljana, Slovenia

**Keywords:** bio-based epoxy adhesives, lignin-based aromatic monophenols, guaiacol, cresol, vanillin, bisphenol A (BPA) replacement

## Abstract

A bio-epoxy surface adhesive for adherence of the metal component species to glass substrate with desirable adhesion strength, converted controlled removal upon request, and bio-based resource inclusion was developed. For the development of resin, three different lignin-based aromatic monophenols, guaiacol, cresol, and vanillin, were used in the chemical epoxidation reaction with epichlorohydrin. The forming transformation process was studied by viscoelasticity, in situ FTIR monitoring, and Raman. Unlike other hydroxyl phenyls, guaiacol showed successful epoxide production, and stability at room temperature. Optimization of epoxide synthesis was conducted by varying NaOH concentration or reaction time. The obtained product was characterized by nuclear magnetic resonance and viscosity measurements. For the production of adhesive, environmentally problematic bisphenol A (BPA) epoxy was partially substituted with the environmentally acceptable, optimized guaiacol-based epoxy at 20, 50, and 80 wt.%. Mechanics, rheological properties, and the possibility of adhered phase de-application were assessed on the bio-substitutes and compared to commercially available polyepoxides or polyurethanes. Considering our aim, the sample composed of 80 wt.% bio-based epoxy/20 wt.% BPA thermoset was demonstrated to be the most suitable among those analyzed, as it was characterized by low BPA, desired boundary area and recoverability using a 10 wt.% acetic acid solution under ultrasound.

## 1. Introduction

Among the more common adhesives, those based on thermosetting polymers known as epoxy resins are widely used, since they are able to provide excellent adhesion to various surfaces and are characterized by versatility, high chemical and thermal resistance, and acceptable mechanical properties [1,2]. Nevertheless, up to 85% of the world’s epoxy resins are based on bisphenol A (BPA), known for its negative impact on the environment due to its estrogenic activity and acute toxicity to freshwater and marine species [3,4,5]. These environmental concerns, working towards the replacement of oil-based chemicals and avoiding the release of toxic materials into our society, are pushing researchers to search for alternative adhesive materials [6].

One of the alternatives for BPA-based epoxies can be extracted from lignocellulosic biomass, which can be easily obtained from low-cost industrial residue (e.g., from the paper industry) and biofuel. Due to its low impact on the environment, inexpensiveness, and abundance, it is also promising for use on an industrial scale. Lignin, in particular, shows good prospects due to its complex polymeric structure rich with a variety of multi-substituted phenolic molecules. These active sites open up possible reaction paths toward epoxidation [7]. On the other hand, the setbacks for the direct use of industrial lignin instead of BPA toward epoxidation can be found in its low reactivity and low solubility in organic solvents, both somewhat dependent on the source of lignocellulosic components and the processing method. The depolymerization of lignin, using procedures such as pyrolysis, causes the disintegration of lignin macromolecules into phenolic compounds with a lower molecular weight, such as guaiacol, methyl guaiacol, and vanillin [8,9]. These lignin-derived molecules are more soluble and more reactive than lignin itself; thus, their phenolic structure with specific functional groups makes them proper candidates for direct glycidylation or epoxidation by epichlorohydrin (ECH).

From the literature, it can be seen that the strength of adhesion of a lignin-based epoxy adhesive strongly depends on its chemical composition, synthesis procedure, and degree of substitution of the adhesive with bio-epoxy. In this manner, adhesives with specific properties can be developed by optimizing the aforementioned conditions. For example, lignin-based epoxy reinforcement materials were tested for their adhesion of an aluminum dolly to a stainless-steel substrate by Ferdosian et al. [10], where depolymerized Kraft and organosolv lignins were used for the synthesis of bio-epoxy resins, which were used for the substitution of BPA-based epoxy in concentrations of 25–100 wt.%. The adhesion strength of the developed epoxies was strongly dependent on the concentration of the bio-epoxy in the mixture; thus, pull-off strengths of 3.9–7.7 MPa were achieved. In another study, vanillin-based bio-epoxy was developed and tested for the adhesion of a metal dolly to an aluminum surface, showing a pull-off strength of 1.37 MPa without reinforcement and 3.71 MPa when reinforced with 2 wt.% calcium nitrate [11]. Another lignin-based epoxy studied for its adhesion properties was based on furfuryl alcohol as a substitute for bisphenol A diglycidyl ether (DGEBA) in concentrations of 0, 10, 20, and 30 wt.%, which was used to attach a dolly to an aluminum substrate. In this case, the adhesion strength increased with the degree of substitution, since the brittleness of DGEBA was reduced. The coating attained a pull-off strength of approximately 1.7 MPa in the case of 30 wt.% substitution [12].

In our work, lignin derivate-based bio-epoxy resins were developed and used to adhere a PVC-based e-component to glass substrates. The compatibility of the adhesive with both the glass substrate and the e-component, as well as its contribution to reduce the harmful impact on the environment, was taken into account. The research was aimed at identifying a bio-epoxy substituted adhesive with specific properties, which were determined by the industrial project partner for the purpose of developing a recyclable glass e-bottle. The strength of the adhesion of the PVC component to the glass substrate was expected to be great enough to allow the handling of the glass substrate, but low enough for its removal in a controlled environment without damaging the glass. In this manner, the target pull-off strength from the glass was approximately 4 MPa, which would allow the removal of the e-component using an acetic acid solution with no visible impact on the substrate. In the first part of the research, three different monophenols were used for the synthesis of bio-based epoxy adhesives, namely, guaiacol, methyl guaiacol, and vanillin. All three monophenols were previously used for the development of bio-based epoxy resins [13,14,15]. The epoxidation of the studied monophenols was monitored using in situ FTIR and Raman analyses. On the basis of the results, the most appropriate monophenol was chosen. In the second part of the research, the reaction conditions, namely, time and NaOH concentration, were varied in order to partially optimize the synthesis of the bio-based epoxy monomer. The synthesized products were characterized and used as partially bio-substituted epoxy adhesives to adhere the metal dolly to a glass substrate. The influence of the degree of substitution was studied, and the bio-based epoxy performance was compared to that of commercially available epoxy and polyurethane adhesives. The strength of the adherence was determined using the pull-off test, while the removal of the adhered metal dolly using an acetic acid solution at low concentrations was also studied.

## 2. Materials and Methods

### 2.1. Materials

Guaiacol (natural; ≥99% FG), methyl guaiacol (≥98%), vanillin (99% Reagent Plus), bisphenol A (97%) and diethylenetriamine (99%, Reagentplus) were purchased from Sigma-Aldrich (St. Louis, MO, USA). Epichlorohydrin (ECH), sodium hydroxide 2-propanol (Emparta ACS) and sodium chloride were obtained from MERCK (Darmstadt, Germany). Glass samples were provided by Steklarna Hrastnik (Hrastnik, Slovenia). Commercial adhesives Araldyte 2028-1 and CHS Epoxy 510 were provided by Henkel (Düsseldorf, Germany). All materials and chemicals were used as received.

### 2.2. Sample Preparation

#### 2.2.1. Synthesis of Bio-Based Epoxy Monomers

**Figure 1:**
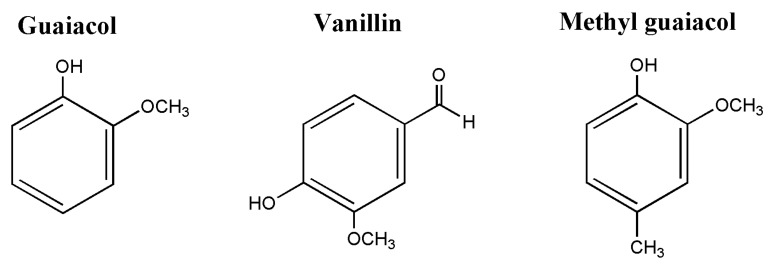
Phenols derived from lignin, used as starting monophenols for epoxy synthesis.

The chosen starting monophenols derived from lignin were guaiacol, vanillin, and methyl guaiacol; their structures are portrayed in Figure 1.

The synthesis of bio-based epoxy molecules was performed according to the altered procedure published by Krol et al. [16]. A 100 mL double-wall glass reactor, equipped with a circulating thermostat, water cooler, FTIR probe, magnetic stirrer, and thermometer, was used. Before the epoxidation reaction, the chosen starting monophenol was dissolved in epichlorohydrin (ECH) in the reactor at 30 °C. Then, 10 min after complete dissolution of the monophenol, 2-propanol was added, and the mixture was stirred for a further 5 min. Next, the temperature was raised to 40 °C, and, 10 min after reaching the plateau, aqueous NaOH was added to initiate O–C bond formation via nucleophilic substitution. Conditions were kept constant during the reaction, and, after the chosen reaction time, the reactor was cooled to room temperature. For comparison, BPA was also used instead of the lignin-based monophenols. After synthesis, the product was extracted and cleaned according to the following procedure: 50 mL of 2 M brine solution was added to the product, the oily phase was separated, and the product was washed again with 2 M brine solution. The newly created oily phase was separated once again and washed two times with distilled water. Afterward, the product was dried in a rotary evaporator system before obtaining the final product. The reaction parameters of all synthesized products are listed in Table 1.

#### 2.2.2. Preparation of the Adhesives

Commercially available epoxy (CHS Epoxy 510, Düsseldorf, Germany) was partly substituted by the prepared bio-epoxy samples at concentrations of 0, 20, 50, and 80 wt.%. Diethylenetriamine (DETA, Sigma-Aldrich, St. Louis, MO, USA) was used as a hardener at a weight ratio of 90:10 (epoxy mixture to hardener). Stabilization after the addition of the hardener was achieved by the addition of 7.845 g of acetone per 1 g of DETA. Additionally, polyurethane adhesive Araldyte 2028-1 was used as a reference. The sample codes and composition of used adhesives are described in Table 2.

#### 2.2.3. Glass Preparation

A glass substrate was provided by Steklarna Hrastnik (model glass Tina 25, Hrastnik, Slovenia). To ensure the repeatability of pull-off tests, the glass substrates were firstly polished with P60 sandpaper in order to remove irregularities, followed by polishing with sandpaper of decreasing roughness (P240–P400–P600–P1200–P2500) to achieve a smooth surface. Polishing was performed using a rotational polisher (Buehler Metaserv 250, Buehler, Lake Bluff, IL, USA) at 400 rpm with the glass in alternating positions (four quadrants). The glass and sandpaper were rinsed with water following each change in polishing direction. Lastly, the substrates were polished using a 3 µm diamond suspension.

#### 2.2.4. Adhesion of the Test Dolly to the Glass Substrate

The aluminum dolly measuring 14 mm in diameter was polished with sandpaper of decreasing roughness (P400–P600–P1200–P2500) using a rotational polisher (Buehler Metaserv 250) at 400 rpm until an even appearance was achieved. The dolly was then dipped in the prepared adhesive and fixated to the bottom of the glass substrates. Curing was performed overnight, at 70 °C in the case of the reference polyurethane adhesive or at 90 °C in the case of the epoxy adhesives.

### 2.3. Analysis and Measurements

#### 2.3.1. Fourier-Transform Infrared (FTIR) Spectroscopy

FTIR analysis was performed in situ, during the epoxidation reaction of the monomers, as well as on the final synthesized monomers; in all instances, the background was subtracted.

For the in situ monitoring, FTIR probe Dicomp AgX, 9.5 mm × 2 m Fiber Silver Halide (Mettler Toledo), was connected to a React IR 45 m (Mettler Toledo, Greifensee, Switzerland) with an inbuilt LN2 NCT detector. The resolution of the FTIR detector was set to 8 cm^−1^ in the iC IR 4.3 software, and 50 scans were taken for each spectrum. The analysis started when the 2-propanol was poured into the reactor and ended when the reaction was completed.

For the final epoxy monomers, FTIR spectra were recorded on a Spectrum Two FTIR (PerkinElmer, Waltham, MA, USA), equipped with a UATR Two detector (PerkinElmer, Waltham, MA, USA). The resolution was set to 8 cm^−1^, and 64 scans were collected per FTIR spectrum.

#### 2.3.2. Raman Spectroscopy and Mapping

A WITec Alpha 300R confocal Raman microscope (WiTec, Ülm, Germany) was used for obtaining the Raman spectra of the epoxy monomers, as well as curing analysis, using a laser wavelength of 532 nm.

#### 2.3.3. Nuclear Magnetic Resonance (NMR) Spectroscopy

^1^H-NMR spectra were recorded on a Bruker 600 MHz NMR spectrometer (Avance Neo 600, Bruker, Billerica, MA, USA), using CDCl_3_ as the solvent and TMS as an internal standard (δTMS = 0.0 ppm).

#### 2.3.4. Rheology

The rheology measurements of PU samples were performed on an Anton Paar MCR301 (Anton Paar, Graz, Austria) under non-isothermal dynamic oscillation profiles. The rheometer was equipped with a parallel geometry of 25 mm. The samples were heated by convection, a solvent trap was used to minimize solvent evaporation, and a temperature control hood was used to prevent heat dissipation. A constant flow of dry nitrogen was applied to eliminate any other chemical processes during heating, and a gap of 0.5 mm was used. Profiles were recorded at a temperature ramp of 2 °C/min, between room temperature and 200 °C, while identifying G’ and G’’. G’ (Pa) is the storage modulus and represents the elastic portion of the viscoelastic behavior. Higher G’ values are related to the majority of the sample being in a “solid” state (G’ > G’’). Its counterpart G’’ (Pa) is the loss modulus and represents the viscoelastic portion of the viscoelastic behavior. Higher G’’ values are related to the majority of the sample being in a “liquid” state (G’’ > G’). The intersect of both curves (G’ = G’’) shows the point at which the material behaves more similarly to a solid than a liquid (i.e., gel point), which indicates the curing point. Therefore, when G’ and G’’ are stable, curing can be regarded as completed. Absolute values give an indication of the rigidity obtained after curing. The influence of the bio-epoxy substitution on G’, G”, and the gel or curing point was examined.

#### 2.3.5. Pull-Off Test

The pull-off test was applied to measure the adhesion force of the glued dollies, using a Positest ATM AT09817 (Defelsko, St. Lawrence, NY, USA). The maximum measurable forces were ~20 MPa.

#### 2.3.6. Removal of the Coating

The sample was ultrasonicated in 10% acetic acid solution at 50 °C until complete removal of the dolly.

## 3. Results

**Figure 2:**
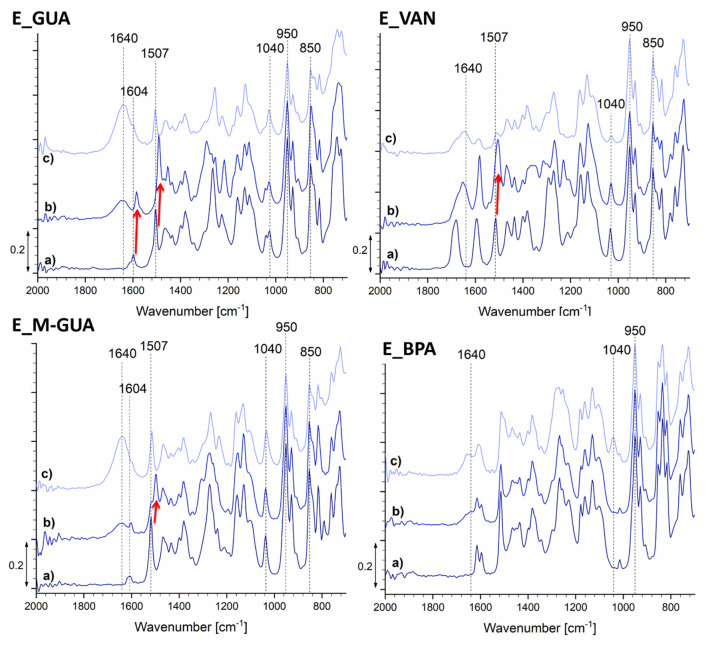
FTIR spectra of the studied samples determined in situ, during the synthesis procedure, where spectra represent the values before the addition of NaOH (a), right after the addition of NaOH (b), and 90 min after the addition of NaOH (c).

An attempt was made to prepare the epoxy resins from the starting monophenols derived from lignin, namely guaiacol, vanillin, and methyl guaiacol, while BPA-based epoxy resin was synthesized for the purpose of comparison. To gain better insight into the reaction of the bio-epoxy resins and successful epoxidation, in situ FTIR was used to analyze the chemical changes during the reaction. The results are portrayed in Figure 2.

Right after the addition of the NaOH, peak shifts were observed in the case of samples E_GUA, E_VAN, and E_M-GUA (marked with red arrows). The first peak shift occurred only for sample E_GUA, from ~1604 cm^−1^ to ~1589 cm^−1^, whereas the second shift occurred for all three mentioned samples, from ~1507 cm^−1^ to ~1492 cm^−1^. After 90 min of reaction time, these peaks shifted back to their original values. These phenomena occurred as a consequence of pH changes. With an increase in the reaction time, samples E_GUA, E_M-GUA, and E_BPA showed an enhancement of the peak at approximately 1040 cm^−1^, indicating the presence of C–O bonds [17], confirming the formation of an epoxy group on the phenol molecule. Furthermore, these samples showed an enhanced peak at 1640 cm^−1^ with increasing reaction time, which could indicate the formation of water as reaction side product [18], although this prediction can only be entirely confirmed using higher recorded wavelengths. The intensity of the enhancement of this peak was the lowest for sample E_BPA, which could be explained by the high viscosity of the reaction mixture, partially preventing the water from coming into contact with the FTIR probe. Furthermore, a decrease in the absorption band at 950 cm^−1^ (O–H bending) [19] occurred, indicating a reduction of the hydroxyl groups on the phenolic compounds. This was in agreement with the desired reaction path and indicated successful epoxidation. Samples E_GUA and E_M-GUA showed a decrease in peak intensity at ~850 cm^−1^ with an increase in reaction time, which can be attributed to the C–Cl bonds in ECH (850–550 cm^−1^) [19]. On the basis of these results, it can be concluded that epoxidation occurred in the case of samples E_GUA, E_M-GUA, and E_BPA. On the other hand, in the case of E_VAN, the peak at 1640 cm^−1^ increased after the addition of NaOH and decreased after 90 min of reaction, implying that the water produced during epoxidation may have entered an unwanted side reaction. Furthermore, this sample showed a decrease in intensity of the peak at 1040 cm^−1^ with an increase in reaction time, which may indicate a reaction of the aldehyde group, possibly with alcohol.

**Figure 3:**
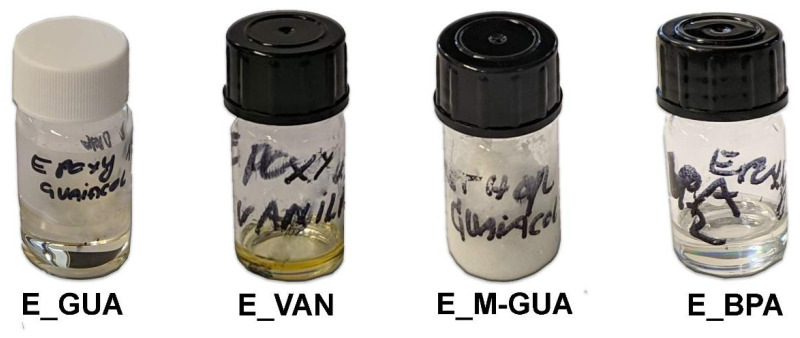
Photographs of the synthesized epoxy resin samples.

The obtained products differed in viscosity, color, and quantity. Photographs of the obtained products are shown in Figure 3, and the visual properties of the finished products are described in Table 3.

The obtained products differed in color, where E_GUA and E_BPA appeared as clear liquids, while E_VAN was dark yellow. The latter also stood out among all samples regarding the quantity of the obtained product, since a lower amount was obtained in comparison to the other samples. Sample E_M-GUA resulted in a solid product at room temperature, which limited its use as an adhesive. The lower viscosity of the guaiacol-based sample indicates that shorter polymer chains were formed when guaiacol was used as a reactant, which could be explained by its simple structure relative to the other lignin-based monophenols.

**Figure 4:**
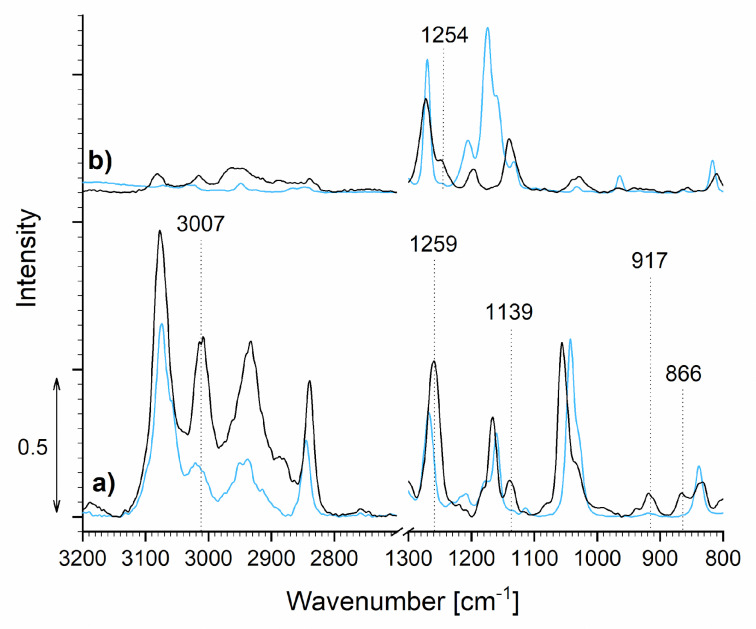
Raman spectra of the starting monophenols GUA (a) and VAN (b) (blue spectra) and synthesized monomers E_GUA (a) and E_VAN (b) (black spectra).

The success of the epoxidation was further studied using Raman spectroscopy. Due to crystallization at room temperature, sample E_M-GUA was not evaluated; thus, only the Raman spectra of E_GUA and E_VAN were studied. The spectra of monophenols GUA and VAN were also added to gain insight into the changes in chemical structure of the samples (Figure 4).

When comparing the spectra of E_GUA and pure GUA, an increase in intensity of the peaks around 3000 cm^−1^ occurred, correlating to the characteristic vibrational peaks of the epoxy group. Moreover, after the reaction, two peaks appeared at 1139 cm^−1^ and 866 cm^−1^, which represent the asymmetric and symmetric stretching C–O–C vibrations of epoxy molecules [20]. At 1259 cm^−1^, an increase in the peak intensity could be seen in the case of E_GUA, representing the epoxy ring vibration, while an increase in the peak at 917 cm^−1^ represents the epoxy ring deformation [21]. The Raman spectrum of E_GUA, therefore, indicates the successful epoxidation of GUA.

On the other hand, the reaction was not successful in the case of sample E_VAN, which confirmed the results obtained from the in situ FTIR analysis. Even though a low-intensity peak representing C–O stretching in an alkyl aryl ether (1254 cm^−1^) [21] appeared, the previously mentioned epoxy-related peaks, representing a successful epoxidation of vanillin, were absent.

**Figure 5:**
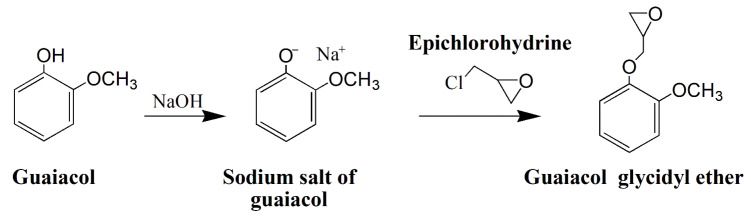
Schematic presentation of the proposed reactions mediating the epoxidation of guaiacol.

On the basis of these results, E_GUA was the only bio-epoxy monomer which was stable at room temperature and underwent the epoxidation reaction. The proposed reaction scheme describing the epoxidation of guaiacol is presented in Figure 5.

Since guaiacol was identified as the most appropriate reagent for the synthesis of bio-epoxy for the partial replacement of BPA, the influence of synthesis parameters on the sample properties was further studied. Accordingly, two new samples were prepared, namely, sample E_GUA_4h, where the time of the synthesis was increased from 1.5 h to 4 h, and sample E_GUA_2NaOH, where the concentration of NaOH used in the synthesis was doubled. A description of the synthesized samples E_GUA_2NaOH and E_GUA_4h is given in Table 4.

Compared to the E_GUA sample, samples E_GUA_4h and E_GUA_2NaOH resulted in a slightly smaller quantity of the finished product. Both samples, E_GUA_4h and E_GUA_2NaOH, appeared slightly yellow.

**Figure 6:**
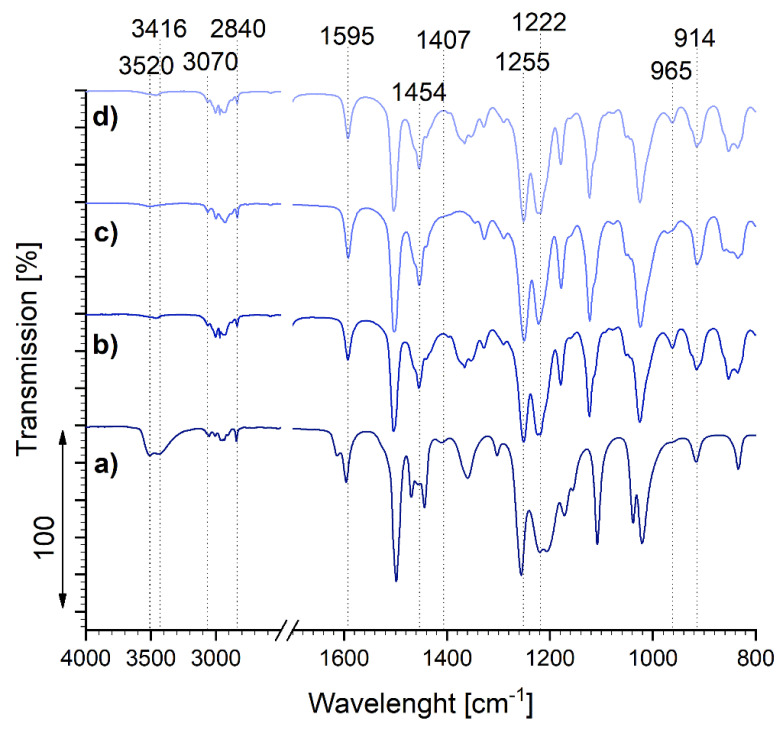
FTIR spectra of pure guaiacol (a) and synthesized epoxy samples E_GUA (b), E_GUA_4h (c), and E_GUA_2NaOH (d).

The synthesized samples based on guaiacol were studied using FTIR, and their spectra were compared to that of pure guaiacol, in order to determine the influence of the synthesis procedure on the chemical properties of the epoxies (Figure 6).

All of the observed samples showed absorption bands in the region of 3070–2840 cm^−1^, corresponding to symmetric and asymmetric C–H stretching in aromatics (~3070 cm^−1^), asymmetric and symmetric –CH_3_ stretching (~2966 cm^−1^ and ~2841 cm^−1^, respectively), and asymmetric –CH_2_ stretching (~2930 cm^−1^) [22,23]. The peaks in the region between 1595 cm^−1^ and 1407 cm^−1^ indicate C = C stretching in aromatics, whereas the bands in the region of 1454–1440 cm^−1^ can be attributed to the stretching and bending vibrations of aliphatic hydrocarbons (CH_3_ and CH_2_) [22,23,24,25]. Absorption bands at 1255 cm^−1^ and 1222 cm^−1^ correspond to C–O stretching in phenolic C–OH and O–CH_3_ groups [22,23,24,25].

Regardless of the synthesis conditions, all of the guaiacol-based samples, E_GUA, E_GUA_4h, and E_GUA_2NaOH, underwent the epoxidation reaction. This was confirmed by the absence of absorption bands at ~3520 cm^−1^ and 3416 cm^−1^ in the epoxy samples, representing the hydroxyl (O–H) groups of monophenols [25,26], which were present in the pure guaiacol sample. Thus, the O–H groups were replaced with epoxy groups during the synthesis. Moreover, with respect to the spectrum of pure guaiacol, the epoxy guaiacol samples exhibited an increase in the intensity of absorption bands around 965 cm^−1^ and 915 cm^−1^, representing epoxide ring vibrations [22].

**Figure 7:**
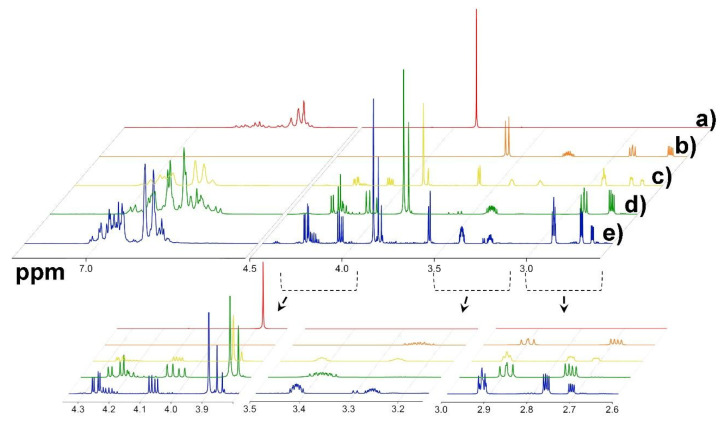
^1^H-NMR spectra of pure guaiacol (a), epichlorohydrin (b), and synthesized guaiacol-based bio-epoxy samples E_GUA (c), E_GUA_4h (d), and E_GUA_2NaOH (e).

The chemical properties of the prepared guaiacol-based epoxies were further determined using ^1^H-NMR analysis. For comparison, the ^1^H-NMR spectra were recorded for pure guaiacol, ECH, and guaiacol epoxies (Figure 7).

^1^H-NMR experiments additionally confirmed the previous assumptions based on the IR analysis, i.e., that an S_N_2 reaction between guaiacol and ECH proceeded, yielding the desired epoxidized product, guaiacol glycidyl ether. Upon analysis, peak shifts and the appearance of additional peaks were observed in the case of all samples (Figure 7(c–e)). In the aromatic region, the peaks shifted to slightly higher values compared to pure guaiacol, whereas the multiplet shape did not change significantly. In the region of 4.5 and 4.8 ppm, two sets of new peaks appeared, corresponding to ArOCH_2_CHO protons in the product. Furthermore, the signal for the OCH_3_ group in the product shifted slightly toward a higher-ppm region, i.e., from 3.87 ppm in guaiacol to 3.88 ppm in the product. Additionally, the OCH_2_CHO multiplet was offset toward a higher-ppm region compared to the multiplet from ECH, whereby a shift from 3.24 to 3.40 ppm was observed. The final two protons in the oxyrane ring (OCH_2_CHOCH_2_) were identified at 2.89 and 2.74 ppm, where the signal at 2.89 ppm of the product overlaid that in unreacted ECH. Additionally, ^1^H-NMR experiments provided insight into the ratio of mixture components in the final isolated products. The biggest difference among reaction isolates (E_GUA, E_GUA_4h, and E_GUA_2NaOH) was in the presence of unreacted ECH, whereby both E_GUA and E_GUA_2NaOH contained a significant amount of unreacted ECH, with the molar ratio between the epoxy product and unreacted ECH being approximately 2:1. On the other hand, only traces of ECH were found in the case of GUA_4h, supporting it as the sample of choice for further investigation.

**Figure 8:**
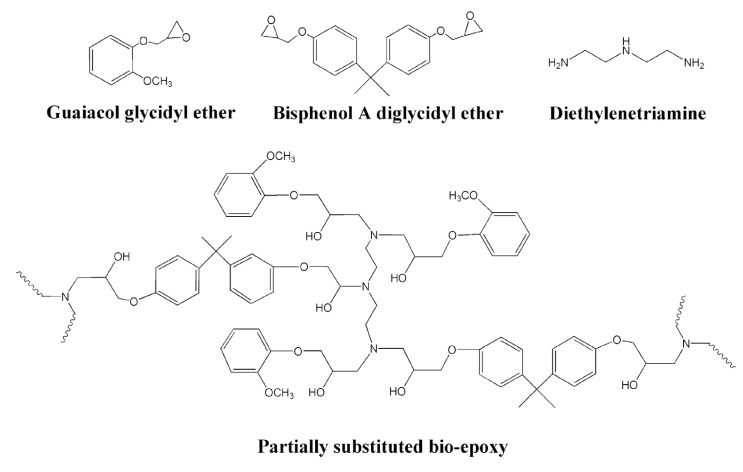
The proposed chemical reaction of guaiacol glycidyl ether, bisphenol A diglycidyl ether (BADGE), and hardener diethylenetriamine yielding the partially substituted bio-epoxy.

In view of the obtained results, sample E_GUA_4h was used for partial substitution of commercially available epoxy adhesive. The proposed chemical reaction of E_GUA_4h (guaiacol glycidyl ether) and E_BPA (bisphenol A diglycidyl ether) with hardener diethylenetriamine to form the partially substituted bio-epoxy adhesive is shown in Figure 8.

Samples composed of BPA epoxy and E_GUA_4h in concentrations of 20, 50, and 80 wt.% were prepared, and the influence of the degree of substitution of the samples on the rheological properties (G’, G”, and the gel point) was examined. The gel point is a significant factor when determining the temperature and time of material hardening. The rheology tests were also performed on reference epoxy and polyurethane adhesives, and the results are presented in Table 5.

Among the studied samples, reference polyurethane adhesive (Ref_PU) possessed the lowest curing temperature and resulted in the hardest material after curing. In the case of the epoxy samples, the curing temperature increased with an increase in the concentration of E_GUA_4h in the epoxy mixture, which rose above 100 °C for the highest (80 wt.%) concentration of bio-epoxy. For the industrial partner, a curing temperature below 100 °C was desirable; thus, despite the gel point of sample 80_bioE being above this value, curing of this sample was possible below the given temperature with an elongation of the curing time.

Next, an aluminum dolly was adhered to the glass substrate using the studied adhesives. On the basis of the rheology results, the Ref_PU adhesive was cured overnight at 70 °C, whereas the epoxy adhesives were cured overnight at 90 °C. The long curing time allowed the successful curing of samples 50_bioE and 80_bioE, despite the lower curing temperature. After the curing, the pull-off test was performed in order to determine the strength of adhesion of the dolly to the glass substrate. Results of the pull-off test are presented in Table 6.

All of the adhesives successfully adhered the dolly to the glass substrate, although the pull-off test portrayed varying pull-off tension values. As previously mentioned, the purpose of this research was not to develop the strongest adhesive, but to develop an adhesive with low BPA content, which could also provide reliable adhesion between glass and metal, as well as easy removal of the metal component under controlled conditions. Thus, the pass or fail threshold was set at a pull-off tension of ~4 MPa.

The reference epoxy adhesive (sample Ref_E) showed the strongest adhesion among the samples and was, therefore, not suitable. Even though the polyurethane reference adhesive (sample Ref_PU) was proven to be the hardest material in the rheology analysis, its adhesion strength was lower than that of the epoxy adhesive, although it was still too strong to achieve the desired results. In the case of both reference adhesives, the glass substrate was damaged upon detachment of the aluminum dolly, which further proved their unsuitability. The pull-off test performed on the samples where partially substituted bio-epoxy was used showed that the adhesion strength of the adhesives could be easily adjusted by changing the degree of substitution. The reason for the reduced adhesion strength of the bio-substituted epoxy samples lies in the reduced crosslinking density of the epoxy adhesive due to the chemical structure of E_GUA_4h. In this manner, sample 20_bioE resulted in strong adhesion, which was comparable to that of the commercially available polyurethane adhesive. Furthermore, when 50 wt.% epoxy was substituted with guaiacol-based bio-epoxy, the pull-off tension decreased drastically and approached the desired value. Surprisingly, compared to sample 50_bioE, the adhesion strength slightly increased in the case of sample 80_bioE, where the bio-epoxy was the prevailing component of the epoxy adhesive. A possible explanation for this phenomenon is the air entrapped in the adhesive. In the case of samples with a lower degree of substitution, the curing time was shorter, whereas sample 80_bioE needed a longer curing time, which resulted in a lower content of entrapped air bubbles and, therefore, greater contact area between the glass and adhesive. Moreover, none of the substituted epoxy adhesives damaged the glass upon forceful removal; thus, detachment of the aluminum dolly was restricted to the adhesive, rather than the substrate.

**Figure 9:**
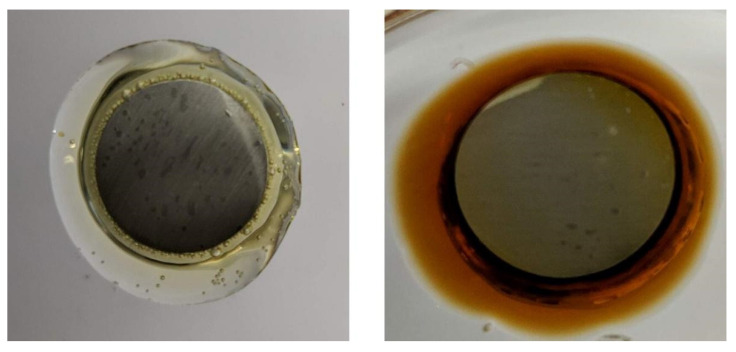
Photos of the aluminum dolly attached to the glass substrate using adhesives 20_bioE (**left**) and 80_bioE (**right**).

According to the results of the pull-off test, sample 80_bioE showed the most desirable properties, since the presence of BPA-based epoxy in the adhesive was drastically decreased and the aluminum dolly could be attached to the glass substrate with the desired adhesion strength. The increased concentration of bio-based epoxy in the adhesive, however, influenced a color change of the sample, which went from transparent to brown with the increase in bio-epoxy concentration, as shown in Figure 9.

An important property of the developed adhesive was easy removal of the e-component from the glass substrate upon request. This property would allow the recyclability of the glass with the attached e-component. For this purpose, experimental removal of the aluminum dolly was performed on the glass substrate with the metal dolly attached using 80_bioE adhesive. The sample was ultrasonicated at 50 °C in a 10% acetic acid solution, which has good compatibility with PVC based materials as well. This method allowed the spontaneous detachment of the metal component from the glass substrate; thus, the developed adhesive successfully demonstrated all of the predetermined properties.

**Figure 10:**
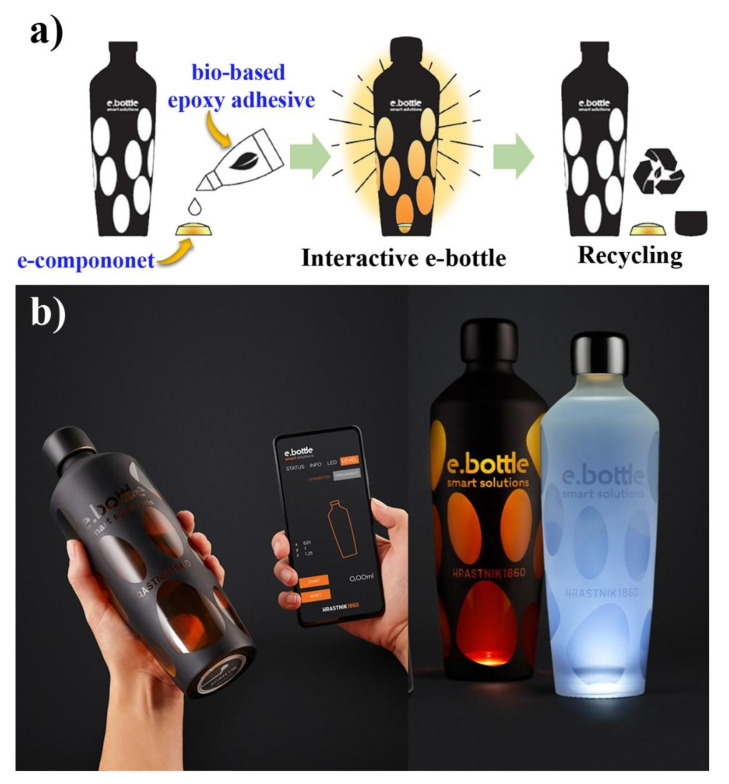
Schematic presentation of the development of an innovative glass e-bottle using the prepared bio-epoxy adhesive (**a**) and pictures of the final product (**b**).

The prepared bio-epoxy adhesive was used by our industrial partner, for the development of a recyclable e-bottle, presented in Figure 10.

## 4. Conclusions

Three lignin-based monophenols, namely, guaiacol, vanillin, and methyl guaiacol, were used for the synthesis of bio-epoxy resins for partial BPA replacement inside an epoxy-based adhesive to achieve the desired adhesive characteristics. The epoxidation reaction was successful in the case of guaiacol and methyl guaiacol; however, the latter resulted in a crystalline product, which limited its use. The influence of the synthesis parameters of the guaiacol-based epoxy was further researched, whereby the synthesis time was increased from 1.5 h to 4 h, and the concentration of NaOH in the synthesis reaction was doubled. Following a comparison of the isolated mixtures, it was determined that a modified procedure with the reaction time increased to 4 h was most appropriate, regarding the purity of the desired product. Therefore, the guaiacol-based epoxy synthesized for 4 h was used for partial substitution of a BPA-based epoxy adhesive in concentrations of 20, 50, and 80 wt.%, and the properties of the adhesives were compared to those of commercially available BPA epoxy and polyurethane adhesives. An increase in the concentration of guaiacol epoxy resulted in an increased gel-point temperature, thus necessitating an increase in temperature or time for successful curing. The adhesion strength of the bio-substituted epoxy adhesive was found to be tunable by varying the degree of substitution of the BPA-based epoxy with the guaiacol-based epoxy. For the intended use of the adhesive, the sample containing 80 wt.% guaiacol epoxy and 20 wt.% BPA epoxy exhibited the most promising results, whereby stable adhesion of metal dolly was achieved along with the potential for its release under controlled conditions. Furthermore, the proposed formulation achieved a drastic decrease in BPA content, which poses an environmental and health hazard. Our bio-based product was successfully tested on an industrial gluing line as a proof of concept to achieve a sustainable e-Bottle product.

## Figures and Tables

**Table 1:** Sample codes and synthesis conditions of the synthesized epoxy monomers.

Sample Name	Starting Phenol	Molar Ratio of the Synthesis Components					Reaction Time (Min)
		Phenol	ECH	NaOH	H_2_O	2-Propanol	
E_GUA	Guaiacol	1	5	1	5	5	90
E_VAN	Vanillin	1	5	1	5	5	90
E_M-GUA	Methyl guaiacol	1	5	1	5	5	90
E_BPA	BPA	1	5	1	5	5	90
E_GUA_2NaOH	Guaiacol	1	5	2	5	5	90
E_GUA_4h	Guaiacol	1	5	1	5	5	240

**Table 2:** Sample codes and composition of the used adhesives.

Sample Code	Concentration of Adhesive Components (wt.%)		
	CHS Epoxy 510	E_GUA_4h	Araldyte 2028-1
Ref_PU	0	0	100
Ref_E	100	0	0
20_bioE	80	20	0
50_bioE	50	50	0
80_bioE	20	80	0

**Table 3:** Properties of the finished products after epoxy synthesis.

Sample	Appearance of the Final Product
E_GUA	Clear slightly yellow liquid of low viscosity
E_VAN	Clear dark-yellow liquid of high viscosity
E_M-GUA	Clear colorless liquid at 40 °C; solid at room temperature
E_BPA	Clear colorless viscous liquid

**Table 4:** Properties of the finished products following guaiacol epoxy resin synthesis.

Sample	Appearance of the Final Product
E_GUA	Clear colorless liquid of low viscosity
E_GUA-4h	Clear light yellow liquid with low to medium viscosity
E_GUA-2NaOH	Clear slightly yellow liquid of low viscosity

**Table 5:** Rheological properties of the studied samples.

Sample	Max G’	Max G”	Max G’−Max G”	Gel Point (°C)
Ref_PU	5.06 × 10^6^	1.00 × 10^5^	4.96 × 10^6^	45
Ref_E	3.23 × 10^6^	8.53 × 10^5^	2.38 × 10^6^	79
20_bioE	1.84 × 10^6^	2.18 × 10^5^	1.62 × 10^6^	94
50_bioE	4.80 × 10^5^	7.87 × 10^4^	4.01 × 10^5^	91
80_bioE	1.67 × 10^5^	2.79 × 10^4^	1.39 × 10^5^	108

**Table 6:** Pull-off tension values of the studied adhesives.

Sample	Pull-Off Tension (MPa)
Ref_PU	10.01
Ref_E	14.05
20_bioE	10.03
50_bioE	3.22
80_bioE	4.81

## Data Availability

The data presented in this study are available in the main text.

## References

[1] Ahmadi Z. (2019). Nanostructured epoxy adhesives: A review. Prog. Org. Coat..

[2] Aliakbari M., Jazani O.M., Sohrabian M., Jouyandeh M., Saeb M.R. (2019). Multi-nationality epoxy adhesives on trial for future nanocomposite developments. Prog. Org. Coat..

[3] Baroncini E.A., Yadav S.K., Palmese G.R., Iii J.F.S. (2016). Recent advances in bio-based epoxy resins and bio-based epoxy curing agents. J. Appl. Polym. Sci..

[4] Kang J.-H., Kondo F., Katayama Y. (2006). Human exposure to bisphenol A. Toxicology.

[5] Vandenberg L.N., Hauser R., Marcus M., Olea N., Welshons W.V. (2007). Human exposure to bisphenol A (BPA). Reprod. Toxicol..

[6] Rodošek M., Mihelčič M., Čolović M., Šest E., Šobak M., Jerman I., Surca A.K. (2020). Tailored Crosslinking Process and Protective Efficiency of Epoxy Coatings Containing Glycidyl-POSS. Polymers.

[7] Hatakeyama H., Hatakeyama T. (2009). Lignin structure, properties, and applications. Biopolymers.

[8] Feghali E., van de Pas D.J., Torr K.M. (2020). Toward Bio-Based Epoxy Thermoset Polymers from Depolymerized Native Lignins Produced at the Pilot Scale. Biomacromolecules.

[9] Pandey M., Kim C.S. (2010). Lignin Depolymerization and Conversion: A Review of Thermochemical Methods. Chem. Eng. Technol..

[10] Ferdosian F., Zhang Y., Yuan Z., Anderson M., Xu C. (2016). Curing kinetics and mechanical properties of bio-based epoxy composites comprising lignin-based epoxy resins. Eur. Polym. J..

[11] Nikafshar S., Zabihi O., Hamidi S., Moradi Y., Barzegar S., Ahmadi M., Naebe M. (2017). A renewable bio-based epoxy resin with improved mechanical performance that can compete with DGEBA. RSC Adv..

[12] Karami Z., Nademi F., Zohuriaan-Mehr M.J., Rostami A. (2017). An efficient fully bio-based reactive diluent for epoxy thermosets: 2-[(Oxiran-2-ylmethoxy) methyl] furan versus a petroleum-based counterpart. J. Appl. Polym. Sci..

[13] Zhao S., Abu-Omar M.M. (2017). Renewable Thermoplastics Based on Lignin-Derived Polyphenols. Macromolecules.

[14] Fache M., Auvergne R., Boutevin B., Caillol S. (2015). New vanillin-derived diepoxy monomers for the synthesis of biobased thermo-sets. Eur. Polym. J..

[15] Hernandez E., Bassett A.W., Sadler J.M., La Scala J.J., Stanzione I.J.F. (2016). Synthesis and Characterization of Bio-based Epoxy Resins Derived from Vanillyl Alcohol. ACS Sustain. Chem. Eng..

[16] Krol P., Krol B., Dziwinski E. (2003). Influence of the synthesis conditions on the properties of low molecular weight epoxy resin. Polimery.

[17] Amarasekara A.S., Garcia-Obergon R., Thompson A.K. (2018). Vanillin-based polymers: IV. Hydrovanilloin epoxy resins. J. Appl. Polym. Sci..

[18] Mojet B.L., Ebbesen S.D., Lefferts L. (2010). Light at the interface: The potential of attenuated total reflection infrared spectroscopy for understanding heterogeneous catalysis in water. Chem. Soc. Rev..

[19] Socrates G. (2001). Infrared and Raman Characteristic Group Frequencies.

[20] Chen X., Chen S., Xu Z., Zhang J., Miao M., Zhang D. (2020). Degradable and recyclable bio-based thermoset epoxy resins. Green Chem..

[21] Vašková H., Křesálek V. (2011). Quasi real-time monitoring of epoxy resin crosslinking via Raman microscopy. Math. Models Methods Appl. Sci..

[22] Maity P., Kasisomayajula S.V., Parameswaran V., Basu S., Gupta N. (2008). Improvement in surface degradation properties of polymer composites due to pre-processed nanometric alumina fillers. IEEE Trans. Dielectr. Electr. Insul..

[23] Phalak G.A., Patil D.M., Mhaske S. (2016). Synthesis and characterization of thermally curable guaiacol based poly(benzoxazine-urethane) coating for corrosion protection on mild steel. Eur. Polym. J..

[24] Azadfar M., Gao A.H., Bule M.V., Chen S. (2015). Structural characterization of lignin: A potential source of antioxidants guaiacol and 4-vinylguaiacol. Int. J. Biol. Macromol..

[25] Yong T.L.-K., Yukihiko M. (2013). Kinetic Analysis of Guaiacol Conversion in Sub- and Supercritical Water. Ind. Eng. Chem. Res..

[26] Cheng H., Wu S., Huang J., Zhang X. (2017). Direct evidence from in situ FTIR spectroscopy that o-quinonemethide is a key intermediate during the pyrolysis of guaiacol. Anal. Bioanal. Chem..

